# Sterility in Women

**Published:** 1880-06

**Authors:** Harry L. Sims


### Selections.

### STERILITY IN WOMEN.

BY HARRY L. SIMS, M. D.

[Read before the San Francisco Obstetrical Society, M rch 2d, 1880.]

*Mr. Chairman and Fellows:* It is not my intention to write an exhaustive article on sterility, with the different methods for its cure as practised by one or another surgeon, but simply to give a few facts and cases that have come under my own observation. Of all the ai ments that have come under my own observation. Of all the ailments that a gyneo'ogist is called upon to treat there is, perhaps, none that he is consulted more than sterility, as it is so often the cause of great unhappiness among individuals, and has led to divorce time and again between man and wife. It is a subject of great interest and has engaged the attention of the profession the world over for ages past; but it is only during the past thirty years that any real progress has been made in the treatment of sterility. McIntosh was the first to begin in the right direction, by dilating the cervical canal with bougies. Then came Sir James Y. Simpson, who followed the same idea by incising the corvix to make the canal permanently open. Pregnacy sometimes followed this incision or d latation, but it will not always cure sterility, by any means.

The sterile condition may be caused by one of many things; there may be a polypus, a fibroid tumor, some malposition, a very painful dysmenorrhea, a contracted cervical canal, or a flexed cervix, or some condition that prevents the semen from passing into the cavity of the uterus. Fully as important a cause for sterility as a flexed cervix, and a rather more frequent one, too, is the existence of an abnormal utero-cervical secretion which poisons or kills the spermatozoa. When we determine that the existing flexure is without doubt the true cause of the sterility, then the operation for incision should be performed; but until we can decide that point for a certainty the operation should not be attempted, otherwise it may be useless and a waste of time.

A valuable, and I may say indispensable, instrument in the diagnosis and treatment of the sterile condition is the microscope, and to my father, Dr. Marion Sims, is due the credit of so simply and clearly proving its usefulness in this respect; for while without the microscope the treatment of sterility is a mere empiricism with it our diagnosis is certain and sure.

In investigating cases of sterility, there are three questions to be determined at the outset, in order to treat our patient scientifically:

1. We must determine whether there are spermatozoa in the semen.

2. We must find out whether the spermatozoa get into the utero-cervical canal.

3. We must ascertain if there are any secretions in the canal which will poison the spermatozoa.

If no spermatozoa are found in the semen, the uterine condition needs no treatment. If spermatozoa do exist in the semen, but do not enter the cervical canal, then arises the question of the propriety of the operation so that they can enter the canal. If, however, the spermatozoa do enter the canal, then, as a rule, no surgical interference will be necessary; and if large numbers of spermatozoa, alive and active, be found there, then the case will need no treatment at all. But should they be found in the canal, and all dead, or nearly so, then it is evident that it is the secretions of the utero-cervical canal which will kill them, and the cause of this abnormal secretion is to be looked for and treated.

It is evident that we must determine these three questions, regardless of any other existing complications, and they can be readily and quickly settled by the microscope. The way I proceed is as follows, when a patient comes to me to be treated for sterility: Having first taken down a history of her case in my note book, I make a careful examination, digitally and with the speculum, to satisfy myself as to the condition and position of the uterus and appendages. Then with a small rubber syringe with a long glass nozzle, the point of which is in the shape of an inverted cone, I take up a small quantity of the secretion found in the vagina near the os uteri and place it upon a clean object glass. I next take a little of the secretion protruding from the os, and then, drawing the uterus forward with a tenaculum, I take still a third sample from as high up as the os internum—all three of which are placed on as many object glasses. The syringe is previously rinsed in moderately warm water, and the point is cone-shaped (a sort of cone within a cone) so that it will hold on to any tenacious mucus more firmly, a difficult thing to get an ordinary point to do. If the case is one of simple stenosis or a flexure, I explain the abnormal condition to the patient, telling her an operation is necessary, but that I cannot decide until I have investigated the case more thoroughly, and perhaps it will take a week or ten days to determine. Having requested her to call again next day, I proceed to examine the specimens under the microscope. If I find spermatozoa in the specimen taken from the os internum I know that that they do enter the uterine cavity, and therefore the sterility is not due to the size of the cervicel canal, and no operation is necessary. If I find them in the vaginal secretion and not at all in the cervical secretions, then I conclude that flexure or contraction is the cause of the sterile state, and that the remedy lies in straightening or enlarging the canal.

But one such microscopic investigation is not be considered conclusive. I repeat it in some cases several times. Sometimes four or five examinations are made, and no spermatozoa found at all. In that case perhaps the vagina expels the semen, as very often happens, as soon as the woman assumes an upright posture. In such a case I explain to the patient and her husband the importance of my investigations, and request that I be allowed to come to their residence in the morning and make the necessary examination. I caution the patient to remain in bed until I come, and tell the husband how necessary it is that coition should take place in the morning before rising. I

have never yet had a patient or her husband become vexed at or refuse to accede to my request, when explained to them in a clear, scientific light, and the reasons given therefor. My office nurse always precedes me to the house and arrange a table for me. Then I take the specimens as before described and examine them at my office as soon as possible.

What I have described is the mode of treating sterility as practiced by my father, Dr. Marion Sims; conclusions arrived at after many years of patient study and experiment. When he first gave his ideas on the subject to the profession, certain journals and physicians, both at home and abroad, condemned in a severe manner his investigating the seminal fluid, and said it was incompatible with decency and self-respect. I cannot see wherein it is any more indelicate or improper to take vaginal or uterine mucus for examination with the microscope than it is to investigate the properties of urine, pus, bile, or feces in a similar manner. It was also said that he cut open cervices recklessly and uselessly; but during the long time I was with my father, I never once knew him to suggest incising the cervix without first most thoroughly investigaing whether or not the operation was perfectly justifiable. When I am satisfied that a case needs no operation, and that the cause of the sterile state is the existing condition of the normal utero-cervical secretions, I at once begin to treat the canal and cavity of the uterus—a treatment which is usually slow, so that patient and doctor sometimes become mutually tired of each other before the cure is effected. The normal secretion of the cervix should be as clear and translucent as the white of an uncooked egg, and when this secretion is full of little snow-white specks it is always inimical to the viability of the spermatozoa. While treating the patient I always examine the cervical mucus every few days. At the beginning, the spermatozoa are all found to be dead. Some time afterwards, as the case progresses, perhaps about one-third will be alive and the rest dead; then, later on, about half dead and the other half alive; and still later, may be nearly all will be alive, with but a few inactive ones; and when, finally, we can take the mucus from the uterus and find the spermatozoa all alive and active thirty hours after coition, we may pronounce the case as cured. But, as I said a while ago, before such a result is arrived at a great deal of patience will be required, both on the patient and the doctor. If, after repeated examinatians of the semen, taken from both cervix and vagina, no spermatozoa at all can be found, then the husband is undoubtedly the cause of the sterile condition, and no treatment to the wife will then be of any use.

The operation of incision of the cervix for sterility is frequently condemned by members of the profession, saying they have done the operation on several occasions, but it was of no benefit. Unfortunately, the decision whether or not to operate, depends with a large proportion of learned and able practitioners, on the uterine sound or probe. If the uterine canal is flexed or contracted, it is advised. If, on the other hand, it enters freely, and without effort, any surgical inteference is discountenanced. In either case the condition of the size and shape of the canal may not be the real cause of the sterile state. The microscope will decide the question of operation or no operation in a very short time, and clearly proves to us what causes the sterility. There is one indisputable fact, and that is that the spermatozoa must reach the uterine cavity in order to produce pregnancy, and they must find a secretion which will not be inimical to their viability. When we have at our command such a valuable help in arriving at a correct and certain diagnosis as the microscope, it is better to trust it than the hap-hazard diagnosis depending on the passage of the sound into the uterine cavity.

Having decided, with the aid of the microscope, that the sterility is due to flexed uterus, an operation is, of course, proposed. We will suppose it is a case of anteflexion. The operation I always perform in such a case is the one originated by my father, and the method of proceeding is as follows:

The patient, being etherized, is turned on the left side, in the semi-prone position, and the Sims speculum introduced. The anterior lip of the cervix is firmly grasped with a tenaculum, and drawn well forward. Then, with the uterome, the posterior lip is incised backwards almost to its intersection with the vagina. Then, with the curved blade of the instrument, the hard gristly band at the os internum is incised interiorly. I next insert my father's large dilator, which is on the principle of a glove stretcher, having three prongs, and thoroughly dilate the incised canal. This stops bleeding almost entirely. Being satisfied that the canal is now sufficiently large, I insert one of the glass or hard rubber plugs, taking care that the plug is not long enough to touch the fundus uteri. To keep the plug in position and prevent bleeding, I tampon the vagina with iron or alum cotton. The former is made by dipping absorbent cotton in a solution of liq. ferri sub-sulph. 1 part in 3 of water, and the latter is a solution of alum, 1 part in 11 of hot water. This cotton is removed piecemeal, a little being taken out each day. The plug is to be taken out on the fourth or fifth day, when the cut surface will be found to have healed beautifully around it. I always order the patient to remain in a recumbent posture, so that there will be no plug slipping downwards. I do not regard the operation as a risky one if proper care is taken. I have performed it many times in the last seven or eight years, and have not had any one die from its effects. I have several times had cellulitis to follow the operation, but with no serious results beyond the inconvenience of a longer stay in bed than was expected. If the case is one of retroflexion, the incisions are simply reversed.

Where the uterus is perfectly straight, with the canal contracted, I make the bilateral incision, and treat it the same as in a flexion. Dr. Emmet objects to the bilateral operation, saying it leads to the same results as lateral laceration in labor, namely, aversion of the lips and ulceration of the eroded surfaces. But in the hundreds of operations I have seen, no such result has ever occurred. The reason these symptoms occur after labor, says Dr. Virgil Hardon, in a recent number of the Boston *Medical and Surgical Journal*, is because the womb is then in a state of fatty degeneration and does not really take on reparative action; besides, at that time, the weight of the organ presses the cervix against the posterior wall of the vagina and forces the lips apart, causing them to roll out as far as the angle of laceration.

I will now relate a few cases.

Mrs. O., aged 28, married six years, sterile: The canal was contracted and the os small and cone-shaped; I found spermatozoa in the utero-cervical canal after the second trial, but they were all dead. The secretion was full of white flakes. Treated the uterus with local application, for four months. She missed the period on the fifth month, went the full term and was delivered of a fine boy.

Mrs. K.: had been married three years and was very anxious for offspring; dreadful dysmenorrhea; uterus anteflexed, very acutely; spermatozoa found in vaginal, but none in cervical secretions. Operated in July last, and she became pregnant in six weeks.

Mrs. S: had been married eight years, with no children; used to suffer horribly with dysmenorrhea; uterus badly anteflexed, and os small and cone-shaped; no spermatozoa found in the utero-cervical canal. Operation proposed and readily acceded to. The uterus was incised antero-posteriorly last June. She is now four months *enciente.*

Mrs. W. married five years and very anxious to have children; had dysmenorrhea, which was followed by an offensive brown discharge; uterus straight; anterior wall somewhat hypertrophied. The os was small. I found spermatozoa in abundance in a drop of mucus from the cervix. Here the question of diagnosis and treatment was settled at once by the microscope. It proved that the spermatozoa did enter the canal and that the secretion there poisoned them, for they were all dead. After three months, living spermatozoa could be found in the cervical mucus thirty hours after coition. She had one more menstrual period after this, when she conceived.

Mrs. H. married three years, sterile; the uterus was of normal size, canal narrow, and os long and cone-shaped. After three days, found spermatozoa in vaginal mucus, but none in cervical. Decided on an operation. The bilateral incision was made. She menstruated three times after the operation, and then became pregnant.

In most of these cases of stenosis and flexed uterus there was severe dysmenorrhea, which the operation invariably relieved. I could go on for an hour and give case after case, but they are all so much alike in symptoms and treatment, that I fear it would become monotonous, and forbear.

The operation for incision of the cervix, as now performed by my father and myself, is a very great improvement over the manner in which we used to perform it three or four years ago. Instead of using the dilator and glass plug, the incised canal was kept open by a cone-shaped pledget of iron cotton, which was removed at the end of three or four days. Sometimes this plug would have to be hastily removed, within twenty-four or forty-eight hours after the operation, on account of a sudden rise in temperature and feverish pulse, preceded by a chill. This was caused by the absorbtion of poisonous matter from the cotton plug, the iron undergoing decomposition and giving rise to a black, offensive-smelling discharge. But this was the exception and not the rule. As now performed, the operation has no such danger, and the using of the dilator makes the straightening, or widening of the uterine canal much more complete. I have also noticed that since the introduction of the dilator and glass plug, the final results of the operation are more satisfactory than before; I mean by that pregnancy more frequently results.

During the past seventeen months, I have operated on nineteen cases of uterine flexions for sterility. Of these cases, thirteen have already resulted in pregnancy, which is a much better percentage than formerly.

DISCUSSION.

Dr. Hutchins remarked that there was a variety of sterility not referred to in the excellent paper of Dr. Sims, namely, sterility attended with or dependent on a membranous exfoliation of the uterine lining membrane. He had seen two such cases. One of them occurred in a patient who, married at 20, had menstruated but once during the year subsequent. The menses then appeared once every four months, the period being attended with discharge of membrane. Electricity was resorted to, and under its use the menses became regular, and the menbrane disappeared. On suspending the electricity, however, the amenorrhea returned, and with it the discharge of membrane. The constant current of 10 to 20 cells was employed.

The President, Dr. Beverly Cole, referred to one point in Dr. Sims' paper which had attracted his particular attention, namely, the semen containing living spermatozoa had been extracted from the cervical canal 30 hours after coition. Not having made the experiment, he was in no position to speak definitely on the point; but he had hitherto been under the impression that spermatozoa could not survive 30 hours. Coste and others establish the fact that the ovule dies within 10 or 12 hours after expulsion from the Graafian vesicle, and thence draw the conclusion that fertilization must take place high up in the Fallopian tube, or perhaps at the ovary itself. But if the ova die in 12 hours, he marvelled that the spermatozoa should live 30 hours, and be then found in the cervix in an active state. He had detected them 8 or 10 hours after coitus, but they were not then very active, and he had inferred that if the ovule died in 12 hours the other element would not survive a much longer period.

Dr. Chismore asked Dr. Sims if, in the course of his investigations, he had found spermatozoa alive 30 hours after coitus.

Dr. Sims replied that he had found them living after 36 hours, and so also had his father. The survival depends in great measure upon the character of the cervical secretion, which, if noxious or acrid, will kill them in a short time.

Dr. Burgess had read somewhere that spermatozoa in certain of the lower animals remain viable 8 or 10 days.

Dr. Whitwell considered the modern method of dilating the uterine canal with the "glove stretcher" instrument, followed by the glass plug, as an improvement on the old operation. The danger of hemorrhage is lessened, and the canal remains patent, and pregnancy more frequently follows.

